# Improving the Management of High-Risk Pregnancies with the Use of the Robson Classification

**DOI:** 10.1055/s-0040-1713910

**Published:** 2020-09-08

**Authors:** Mario Dias Correa Junior, Bárbara Moreira Ribeiro Trindade dos Santos, José Reinaldo Correa Roveda, Laura Carolina Menezes Vieira Silva, Larissa Silva Guimarães, Samuel Cristóvão Lopes Gonçalves

**Affiliations:** 1 Faculdade de Medicina, Universidade Federal de Minas Gerais, Belo Horizonte, MG, Brazil; 2 Medical School, Centro Universitário de Belo Horizonte, Belo Horizonte, MG, Brazil

**Keywords:** robson classification, hospital management, maternity, cesarean section, evidence-based clinical practice, birthing centers, classificação de robson, gestão hospitalar, maternidade, cesariana, prática clínica baseada em evidências, maternidades

## Abstract

**Objective** To analyze the rates of cesarean delivery longitudinally in a university hospital using the Robson classification.

**Methods** Data related to births performed between 2014 and 2018 and recorded in the Maternal and Neonatal Health Information System (Sistema de Informações em Saúde Materna e Neonatal, SISMATER, in Portuguese) were analyzed using the Robson classification. As an aid, we used articles published in the last five years that approach the same topic in other Brazilian maternity hospitals; they were retrieved from the LILACS, MEDLINE, CINAHL, Scopus, Web of Science and Cochrane Library databases.

**Results** There was little variation in the total rate of cesarean sections in the period; however, the profile of each group changed over the years. It was possible to verify a significant reduction in the participation of groups of pregnant women with lower risk and an increase in high-risk pregnancies, attributable to the decrease in beds in the institution, with a greater transfer of patients. In addition, there was a reduction in cesarean sections among the lower-risk groups, while the rate among the higher-risk groups remained stable.

**Conclusion** The use of the Robson classification to stratify cesarean deliveries contributes to a better analysis of the indications for cesarean delivery, enabling the establishment of strategies to reduce the rates, generating a positive impact on hospital management and quality of care.

## Resumo

**Objetivo** Analisar longitudinalmente as taxas de parto cesáreo em um hospital universitário usando a classificação de Robson.

**Métodos** Foram analisados, por meio da classificação de Robson, dados relacionados a partos realizados entre 2014 e 2018 e armazenados no Sistema de Informações em Saúde Materna e Neonatal (Sismater). Para auxílio, foram utilizados artigos publicados nos últimos cinco anos que abordavam o mesmo tema em outras maternidades brasileiras, tendo como fonte as bases de dados LILACS, MEDLINE, CINAHL, Scopus, Web of Science e Cochrane Library.

**Resultados** A taxa total de cesárea variou pouco no período; no entanto, alterou-se o perfil de cada grupo ao longo dos anos. Foi possível constatar redução significativa da participação de grupos de contendo gestantes de risco habitual e aumento das gestações de alto risco, atribuíveis à diminuição de leitos na instituição, com maior transferência de pacientes. Além disso, houve uma redução na taxa de cesáreas nos grupos de mais baixo risco, enquanto a taxa dos grupos de risco mais elevado se manteve estável.

**Conclusão** A utilização da classificação de Robson para estratificar os partos cesáreos contribui para uma análise melhor das indicações do parto cesáreo, o que permite o estabelecimento de estratégias para a redução das taxas, gerando um impacto positivo na gestão hospitalar e na qualidade assistencial.

## Introduction

The first record of a cesarean section (CS) in a live parturient dates back to around 1500, and previously CSs were performed only after maternal death, to save the fetus. Over time, this surgery has improved and is currently considered safe, but it still presents risks for the mother and the newborn. 1 By definition, CS is a procedure in which the fetus is removed through an abdominal incision. Such practice, however, does not provide justifiable benefits from the point of view of maternal and newborn health when not indicated, presenting more risks than potential benefits.

What is observed in the world is a tendency to increase the number of CSs. Data published by the World Health Organization (WHO) shows that the percentage rose from 6.7% to 19.1% between 1990 and 2014, in an analysis performed in 121 countries. Brazil had CS rates close to 37.8% 2 in 1994, and in 2014, this type of procedure was performed in 53.88% of the cases. 2 Since 1985, the WHO suggests that CS rates remain between 10% and 15%. 3 From a critical point of view, it can be said that this objective is unattainable in certain populations, since it does not take into account the social realities of geosocial stratification.

Although the rate of CS is a good indicator of the access to health services, it is important to emphasize that, from a financial point of view, normal delivery is much more cost-effective, and it presents lower risks when well indicated. In addition, the Brazilian government pays the costs of the unnecessary CSs that occur in the public system; therefore, to aim for a better quality of care and a more efficient resource management is fundamental to question the excess of CSs.

In this regard, the WHO, in 2015, suggested the adoption of the Robson classification as a stratification of CSs according to current and previous gestational data in which all pregnant women fit. 3 This categorization is based on six characteristics: gestational age (term, preterm); obstetric history (nulliparous or multiparous); presence of uterine scar (anterior CS); number of fetuses (single or multiple gestation); fetal presentation (cephalic, pelvic or abnormal); and the way in which the birth developed (spontaneous, induced, CS by choice). This method generates 10 mutually-exclusive and fully-inclusive groups (three of them are also divided into two subgroups), enabling a longitudinal analysis of the pattern for the indication of CSs in each of the selected groups. 4

In addition to the study from an assistance point of view, it is possible to use the Robson classification to promote strategies on each specific group, making the management methods more objective. Thereby, through the aforementioned stratification, we aimed to longitudinally analyze the CS rates in Hospital das Clínicas, Universidade Federal de Minas Gerais (HC-UFMG). By understanding the variations in these rates, it is possible to determine in which groups we should act to reduce CS rates and also provide subsidies for the improvement of the quality of care, clinical-obstetric outcomes, and the allocation of public resources to a more qualified and humanized service.

## Methods

To carry out the study, we used the set of data related to all deliveries that took place in HC-UFMG between 2014 and 2018 that were recorded in the Maternal and Neonatal Health Information System (Sistema de Informações em Saúde Materna e Neonatal, SISMATER, in Portuguese). Of the total, 99.3% could be classified in the Robson classification, and the rest were discarded from the present study. The data obtained were analyzed using the Robson classification, with the main objective of performing a longitudinal analysis of CS rates. To carry out the analysis, we searched in the specialized literature articles published between 2015 and 2019 that dealt with the use of the Robson classification in Brazilian maternity hospitals in the following databases: BVS-LILACS (via Bireme), MEDLINE (via PubMed), CINAHL, Scopus, Web of Science and the Cochrane Library. The following descriptors were used: *cesarean deliveries* (and similar descriptors) together with *epidemiology* , *incidence* and *prevalence* , as well as *Robson classification* along with *epidemiology* , *incidence* , and *prevalence* . Only the year of publication and the languages (Portuguese, English and Spanish) were used as filters.

## Results

**Fig. 1:**
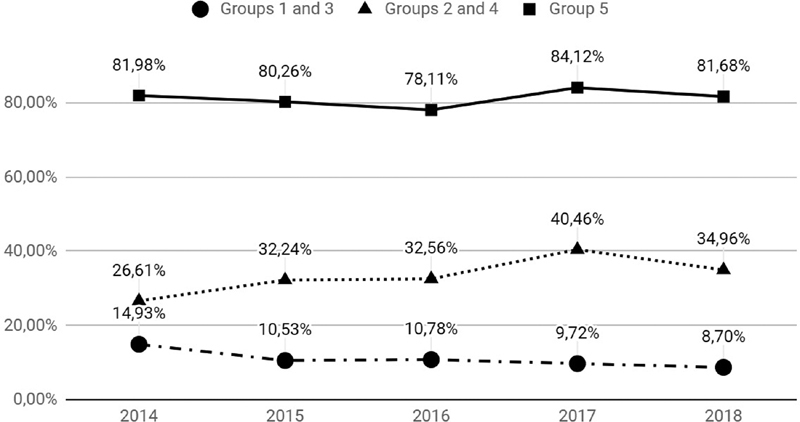
Comparative analysis of the rate of cesarean section between “groups 1 and 3,” “groups 2 and 4” and “group 5”.

**Fig. 2:**
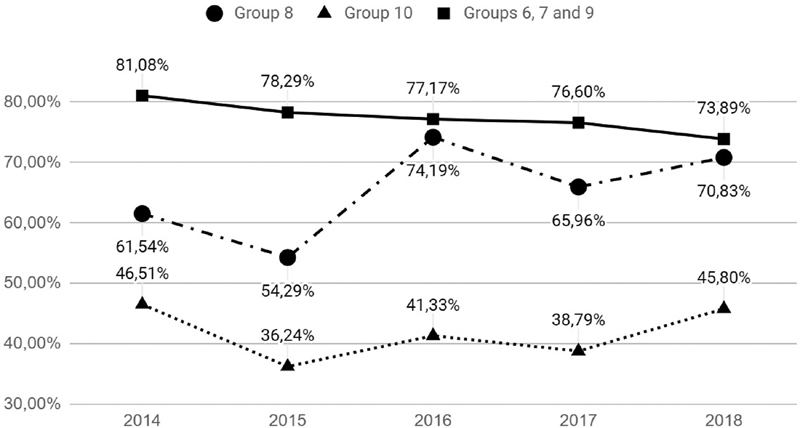
Comparative analysis of the rate of cesarean section between “group 8,” “group 10” e “groups 6, 7 and 9”.

Between 2014 and 2018, there were 10,356 deliveries, 10,284 of which were analyzed according to the Robson ten-group classification. Groups 2, 4 and 5 were not analyzed with the subdivisions due to the fact that the system in which the data was analyzed is from 2013, and, therefore, prior to the current recommendations. The CS rates showed a small variation in the period (37.1 ± 2.6%; Table 1 ); however, the profile of each group changed throughout the years ( Table 2 ). There was a significant decrease in the number of CSs in groups 1 and 3 (from 14.9% to 8.7%). This represents a considerable change, since these groups are usually composed of low-risk patients. In groups 2 and 4, in which the patients were submitted to induction of labor or CS before the beginning of labor, there was an increase in the rate from 26.6% to 34.9%, demonstrating that a better analysis should be performed in these groups to identify the causes of this increase. In group 5,–patients with previous CS –, the rates remained unaltered during the period: 81%, which, however, are high rates, indicating that the implementation of effective strategies to encourage vaginal birth after CS (VBACS) may be effective ( Fig. 1 ). Groups 6, 7 and 8, which are of pelvic and transverse presentation, presented a drop, ranging from 83.1% to 71.9%. The rates in multiple pregnancies (group 8) went from 61.5% to 70.8%, and in preterm deliveries they remained constant, ranging from 46.5% to 45.8% ( Fig. 2 ).

**Table 1:** Number of cesarean sections in relation to the total number of births per Robson group

Robson classification	2014		2015		2016		2017		2018	
	Total number of deliveries	Number (%) of cesarean sections	Total number of deliveries	Number (%) of cesarean sections	Total number of deliveries	Number (%) of cesarean sections	Total number of deliveries	Number (%) of cesarean sections	Total number of deliveries	Number (%) of cesarean sections
Total deliveries	2,110	761 (36.1)	2,110	730 (34.6)	1,993	715 (35.9)	2,161	844 (39.1)	1,982	785 (39.6)
Group 1	507	116 (22.9)	384	59 (15.4)	372	65 (17.5)	352	53 (15.1)	275	42 (14.9)
Group 2	229	69 (30.1)	293	116 (39,6)	319	130 (40.8)	322	159 (49.4)	298	128 (43.0)
Group 3	451	27 (6)	442	28 (6.3)	361	14 (3.9)	420	22 (5.2)	369	15 (4.1)
Group 4	158	34 (21.5)	194	41 (21.1)	200	39 (19.5)	244	70 (28.7)	234	58 (24.8)
Group 5	333	273 (82)	309	248 (80.3)	297	232 (78.1)	340	286 (84.1)	333	272 (81.7)
Group 6	52	43 (82.7)	53	42 (79.2)	60	45 (75.0)	60	46 (76.7)	80	59 (73.8)
Group 7	45	34 (61.)	67	50 (74.6)	61	48 (78.7)	69	52 (75.4)	71	52 (73.2)
Group 8	52	32 (61.5)	35	19 (54.3)	31	23 (74.2)	47	31 (66.0)	48	34 (70.8)
Group 9	14	13 (92.9)	9	9 (100.0)	6	5 (83.3)	12	10 (83.3)	6	5 (83.3)
Group 10	258	120 (46.5)	298	108 (36.2)	271	112 (41.3)	281	109 (38.8)	262	120 (45.8)
Total number of cesarean sections classified	2,099	761 (36.3)	2,084	720 (34.5)	1,978	713 (36)	2,147	838 (39)	1,976	784 (39.7)

**Table 2:** Relative contribution of each Robson group to the total number of cesarean sections

Robson classification	Participation of each group in the total number of cesarean sections				
	2014 (%)	2015 (%)	2016 (%)	2017 (%)	2018 (%)
Group 1	15.20	8.20	9.10	6.30	5.20
Group 2	9.10	16.10	18.20	19.00	16.30
Group 3	3.50	3.90	2.00	2.60	1.90
Group 4	4.50	5.70	5.50	8.40	7.40
Group 5	35.90	34.40	32.50	34.10	34.70
Group 6	5.70	5.80	6.30	5.50	7.50
Group 7	4.50	6.90	6.70	6.20	6.60
Group 8	4.20	2.60	3.20	3.70	4.30
Group 9	1.70	1.30	0.70	1.20	0.60
Group 10	15.80	15.00	15.70	13.00	15.30
Total number of cesarean sections classified	761	720	713	838	784

Regarding the size of each group, groups 1 and 3 decreased from 45.6% to 32.6%, showing that there was a reduction in the number of patients admitted with spontaneous labor, while groups 2 and 4 (induction or elective CS) increased from 18.4% to 26.9%. These variations are attributed to the reduction in hospital beds in the institution, with greater transference of low-risk patients, giving priority to the hospitalization of more severe patients.

Group 5, in which the women already had a CS, has a high participation rate that has small changes (35.9% and 34.7%) ( Table 2 ). However, the CS rate is around 80% – the recommended rate is between 50% and 60% –, which reflects an obstetric fear, since evidence shows that 70% of vaginal deliveries with previous history of CS have a good evolution, but this group has a higher chance of uterine rupture, which is considered an obstetric emergency.

Groups 6 and 7, which are the ones of pelvic presentation, showed an increase in the rate of CS, which changed from 5.7% to 7.5% and from 4.5% to 6.6% respectively, which can be considered a reflection of a greater difficulty in transferring patients with an indication for an elective CS to other services. The rates of the multiple-gestation (group 8) and preterm-birth (group 10) groups were constant, ranging from 4.2% to 4.3% and 15.8% to 15.3%, while the gestations with abnormal positions (group 9) showed a reduction from 1.7% to 0.6% ( Table 3 ).

**Table 3:** The Robson ten-group classification

Group 1	Nulliparous, singleton, cephalic, ≥37 weeks of gestation, in spontaneous labor.	
Group 2	Group 2a	Nulliparous, singleton, cephalic, ≥37 weeks of gestation with induced labor.
	Group 2b	Nulliparous, singleton, cephalic, ≥37 weeks of gestation with caesarean section before labor.
Group 3	Multiparous (excluding previous cesarean section), singleton, cephalic, ≥37 weeks of gestation, in spontaneous labor.	
Group 4	Group 4a	Multiparous without a previous uterine scar, singleton, cephalic pregnancy, ≥37 weeks of gestation, induced.
	Group 4b	Multiparous without a previous uterine scar, with singleton, cephalic pregnancy, ≥37 weeks of gestation with cesarean section before labor.
Group 5	Group 5.1	Singleton, cephalic, ≥37 weeks of gestation, with one previous cesarean section.
	Group 5.2	Singleton, cephalic, ≥37 weeks of gestation, with more than one previous cesarean section.
Group 6	All nulliparous with a single breech.	
Group 7	All multiparous with a single breech (including previous cesarean section).	
Group 8	All multiple pregnancies (including previous cesarean section).	
Group 9	All women with a single pregnancy in transverse or oblique lie (including those with previous cesarean section).	
Group 10	All singleton, cephalic, < 37 weeks of gestatio (including previous cesarean section).	

Source: Robson (2001). 5

## Discussion

In 2001, Dr. Michael Robson published a study 5 classifying the indications for CS at the National Maternity Hospital in Dublin, Ireland, and his classification was standardized by the WHO in 2015. 6 Since then, it has been used as a protocol first in European countries and, after that, it has been applied in poorer countries in an effective way all around the world. 7 8

In Brazil, specialty hospitals created their own protocols based on the Robson classification to monitor their indications for CS as well as to effectively control the financial outcomes. 9 10 In Brazil, the WHO recommendation was applied to maternity hospitals through the Adequate Childbirth Program – which was proposed by the National Agency for Supplementary Health (Agência Nacional de Saúde Suplementar, in Portuguese) along with Hospital Albert Einstein and the Institute for Healthcare Improvement –, which aims to reduce the CS rates – and the Apice On Project – which was developed by the Nursing School at UFMG with 95 institutions –, whose goal is the humanization of childbirth in the Brazil regarding the basic training of professionals against the rising morbimortality indicators. 11 12 13

At the Otto Cirne Maternity of HC-UFMG, the non-subdivision of groups 2, 4 and 5 enabled a better analysis of the risk stratification. Despite that, the management project was able to help reduce the CS rates in low-risk patients (groups 1 and 3), which shows a good alignment to the WHO standards, since they indicate that professionals are adapting to the indications for CS. Even though our CS rate has remained constant, this is due to the type of patients who seek the hospital (high-risk reference), and due to the years with a high CS rate, which results in many patients with previous CS (group 5), which is a possibility of change since in many institutions the VBACS has been offered with a consequent decrease in this group as well.

This classification has shown to be really important in the health management of our maternity hospital, because it has enabled us to obtain knowledge regarding the profile of the patients due to the size of each group. Moreover, it has enabled the observation of the indications for CS, mainly in order for us to make corrections and adjustments. Ultimately, the Robson classification enables the comparison of our data with that of other different maternity hospitals.

## Conclusion

The classification of CSs into subgroups as recommended by the WHO through the Robson classification contributes to a better analysis of the indications for CS, and it also helps to improve the quality of care. Thus, a positive impact on hospital management can be observed, enabling the direct reduction in costs, with better allocation of the budget, with a reduction in expenses regarding non-indicated procedures, and indirectly, with shorter hospitalization time and lower complications involving secondary costs. The present work corroborates the argument that the use of the Robson classification improves the management of the procedures performed in maternity wards, both regarding the assessment of the indication for CS and the continuous revision of the protocols adopted. Thus, the present study shows that there are many future opportunities related to the use of the Robson classification that are applicable to maternity management, something as necessary as desired to positively impact the health of women and children and to direct resources to make health services more accessible and unbiased.

## References

[1] Rezende J M A primeira operação cesariana em parturiente viva São Paulo Editora Unifesp 2009 171 172

[2] Ministério da Saúde. DATASUS Indicadores e Dados Básicos - Brasil – 2012 [Internet] 2013[cited 2019 Jun 8]. Available from:http://tabnet.datasus.gov.br/cgi/idb2012/matriz.htm

[3] Organização Mundial da Saúde Declaração da OMS sobre taxas de cesáreas [Internet] 2015[cited 2019 Jun 8]. Available from:https://apps.who.int/iris/bitstream/handle/10665/161442/WHO_RHR_15.02_por.pdf?sequence=3

[4] World Health Organization Robson Classification: implementation manual [Internet] Geneva WHO 2017[cited 2019 Jun 8].https://apps.who.int/iris/bitstream/handle/10665/259512/9789241513197-eng.pdf?sequence=1

[5] Robson M S Classification of caesarean sections Fetal Matern Med Rev 2001 12 01 23 39. Doi: 10.1017/S0965539501000122

[6] Boatin A A Cullinane F Torloni M R Betrán A P Audit and feedback using the Robson classification to reduce caesarean section rates: a systematic review BJOG 2018 125 01 36 42. Doi: 10.1111/1471-0528.14774 28602031 10.1111/1471-0528.14774 PMC5763379

[7] Triunfo S Ferrazzani S Lanzone A Scambia G Identification of obstetric targets for reducing cesarean section rate using the Robson Ten Group Classification in a tertiary level hospital Eur J Obstet Gynecol Reprod Biol 2015 189 91 95. Doi: 10.1016/j.ejogrb.2015.03.030 25898370 10.1016/j.ejogrb.2015.03.030

[8] Yadav R G Maitra N Examining cesarean delivery rates using the Robson's Ten-group Classification J Obstet Gynaecol India 2016 66 01 1 6. Doi: 10.1007/s13224-015-0738-1 10.1007/s13224-015-0738-1 PMC5016390 27651569

[9] Ferreira E C Pacagnella R C Costa M L Cecatti J G The Robson ten-group classification system for appraising deliveries at a tertiary referral hospital in Brazil Int J Gynaecol Obstet 2015 129 03 236 239. Doi: 10.1016/j.ijgo.2014.11.026 25704253 10.1016/j.ijgo.2014.11.026

[10] Nakamura-Pereira M do Carmo Leal M Esteves-Pereira A P Use of Robson classification to assess cesarean section rate in Brazil: the role of source of payment for childbirth Reprod Health 2016 13 03 128. Doi: 10.1186/s12978-016-0228-7 27766941 10.1186/s12978-016-0228-7 PMC5073850

[11] Agência Nacional de Saúde Suplementar, Sociedade Beneficente Israelita Brasileira Hospital Albert Einstein, Institute for Healthcare Improvement Cartilha nova organização do cuidado ao parto e nascimento para melhores resultados de saúde: Projeto Parto Adequado - fase 1 [Internet] Rio de Janeiro ANS 2016[cited 2019 Jun 9]. Available from:http://www.ans.gov.br/images/stories/Materiais_para_pesquisa/Materiais_por_assunto/web_total_parto_adequado.pdf

[12] Ministério da Saúde. Secretaria de Atenção à Saúde Apice on: aprimoramento e inovação no cuidado e ensino em obstetrícia e neonatologia [Internet] Brasília (DF) Ministério da Saúde 2017[cited 2019 Jun 9]. Available from:https://portalarquivos.saude.gov.br/images/pdf/2017/agosto/18/Apice-On-2017-08-11.pdf

[13] Mascarello K C Horta B L Silveira M F Maternal complications and cesarean section without indication: systematic review and meta-analysis Rev Saude Publica 2017 51 105. Doi: 10.11606/s1518-8787.2017051000389 29166440 10.11606/S1518-8787.2017051000389 PMC5697917

